# Comparative Analysis of Salt Responsive MicroRNAs in Two Sweetpotato [*Ipomoea batatas* (L.) Lam.] Cultivars With Different Salt Stress Resistance

**DOI:** 10.3389/fpls.2022.879819

**Published:** 2022-07-07

**Authors:** Zhengmei Yang, Tingting Dong, Xibin Dai, Yiliang Wei, Yujie Fang, Lei Zhang, Mingku Zhu, Ghazala Nawaz, Qinghe Cao, Tao Xu

**Affiliations:** ^1^Jiangsu Key Laboratory of Phylogenomics and Comparative Genomics School of Life Sciences, Jiangsu Normal University, Xuzhou, China; ^2^Department of Applied Biology, Chonnam National University, Gwangju, South Korea; ^3^Jiangsu Xuzhou Sweetpotato Research Center, Xuzhou, China; ^4^Jiangsu Key Laboratory of Crop Genomics and Molecular Breeding, Yangzhou University, Yangzhou, China; ^5^Department of Botanical and Environmental Sciences, Kohat University of Science and Technology, Kohat, Pakistan

**Keywords:** sweetpotato, microRNA, salt stress, high-throughput deep sequencing, degradome sequencing

## Abstract

Sweetpotato [*Ipomoea batatas* (L.) Lam.] is an important food, vegetable and economic crop, but its productivity is remarkably affected by soil salinity. MiRNAs are a class of endogenous non-coding small RNAs that play an important role in plant resistance to salt stress. However, the function of miRNAs still remains largely unknown in sweetpotato under salt stress. Previously, we identified salt-responsive miRNAs in one salt-sensitive sweetpotato cultivar “Xushu 32.” In this study, we identified miRNAs in another salt-tolerant cultivar “Xushu 22” by high-throughput deep sequencing and compared the salt-responsive miRNAs between these two cultivars with different salt sensitivity. We identified 687 miRNAs in “Xushu 22,” including 514 known miRNAs and 173 novel miRNAs. Among the 759 miRNAs from the two cultivars, 72 and 109 miRNAs were specifically expressed in “Xushu 32” and “Xushu 22,” respectively, and 578 miRNAs were co-expressed. The comparison of “Xushu 32” and “Xushu 22” genotypes showed a total of 235 miRNAs with obvious differential expression and 177 salt-responsive miRNAs that were obviously differently expressed between “Xushu 32” and “Xushu 22” under salt stress. The target genes of the miRNAs were predicted and identified using the Target Finder tool and degradome sequencing. The results showed that most of the targets were transcription factors and proteins related to metabolism and stress response. Gene Ontology analysis revealed that these target genes are involved in key pathways related to salt stress response and secondary redox metabolism. The comparative analysis of salt-responsive miRNAs in sweetpotato cultivars with different salt sensitivity is helpful for understanding the regulatory pattern of miRNA in different sweetpotato genotypes and improving the agronomic traits of sweetpotato by miRNA manipulation in the future.

## Introduction

Soil salinization is an increasingly serious worldwide agricultural problem that restricts crop production and reproduction. Salt stress affects photosynthesis, signal transduction, protein synthesis, energy and lipid metabolism through osmotic stress, ionic stress and secondary stress (Hurkman and Tanaka, 1987; Zhu, 2002; Hnilickova et al., 2021; Guo et al., 2022). Thus, salt stress hinders seed germination, delays growth, slows tissue and organ differentiation, causes dwarf morphology and reduces yield (Van Zelm et al., 2020; Liu et al., 2021). Plants can establish salt resistance and/or tolerance at the morphological, physiological, biochemical, cellular and molecular levels to cope with salt stress through osmoregulation, hormone regulation, antioxidant enzyme, photosynthetic pathway change, protein regulation and other ways (Yu et al., 2020). Meanwhile, plants respond to salt stress by regulating the RNA modification and gene expression of salt stress-related genes, which are involved in reactive oxygen species homeostasis, osmotic adjustment, channel protein synthesis, antioxidant and signal transduction (Van Zelm et al., 2020; Yu et al., 2020; Hu et al., 2021). The regulation of these protein-coding sequences and non-coding RNA also plays an important role in stress tolerance (Zhang et al., 2007).

MicroRNA (miRNA) is an endogenous small RNA that widely exists in plants and plays an important role in the regulation of gene expression at the transcription, post-transcription and translation levels. Mature miRNA combines with AGO1 and other proteins to form a miRNA-induced silencing complex, which can cleave the target gene or inhibit the translation of the target gene. At present, miRNAs and their target genes related to salt stress have been identified in many plants, such as broccoli (*Brassica oleracea*), *Populus tomentosa*, radish (*Raphanus sativus*) and maize (*Zea mays*) (Dong et al., 2009; Ren et al., 2013; Tian et al., 2014). MiRNA enables plant to adapt to salt by regulating salt stress-related genes (Xu et al., 2021). Overexpression of miR156a decreased the salt tolerance of apple plant, while overexpression its target gene *MdSPL13* improved salt tolerance (Ma et al., 2020). Overexpression of the target gene *PeNAC070* of miR164 promoted lateral root development, delayed stem elongation, and increased sensitivity of transgenic *Arabidopsis* plants to salt stresses (Lu et al., 2017). Overexpression of the target gene *GmNFYA3* of miR169 increases sensitivity to high salinity in *Arabidopsis* (Ni et al., 2013). Salt stress induces miR319 expression, which can increase plant tolerance to salt stress by down-regulating its target gene (Zhou et al., 2013). Overexpression of Osa-miR393a in creeping bentgrass can improve the salt tolerance of transgenic plants by down-regulating its target genes *AsAFB2* and *AsTIR1* (Zhao et al., 2019). Overexpression of miR394 in *Arabidopsis* can cause hypersensitivity under salt stress, whereas the overexpression of the target gene *LCR* of miR394 enhances its tolerance to salt stress; therefore, miR394 can negatively adjust the salt stress adaption of plants (Song et al., 2013). Overexpression of osa-MIR396c decreases salt stress tolerance (Gao et al., 2010). Constitutive expression of rice microRNA528 enhances tolerance to salinity stress and nitrogen starvation in creeping bentgrass (Yuan et al., 2015). These results suggested that microRNAs could be used as a powerful tool for improving salinity resistance in crops (Xu et al., 2021).

As one of the most important and promising crops in the world, sweetpotato (*Ipomoea batatas* (L.) Lam.) has an indispensable role in reducing poverty and promoting food safety (Wan et al., 2020; Zhang et al., 2020; Li et al., 2022). Sweetpotato has significant nutritional value for humans, because it contains a variety of nutrients, including protein, anthocyanins, minerals, carotenoids, dietary fiber and vitamins (Zhu et al., 2020; Sun H. et al., 2022). However, salt stress has great adverse effects on the growth, fresh weight, health-promoting compounds and antioxidant activity of sweetpotato. Therefore, the study on the mechanism of salt tolerance in sweetpotato is very important for developing salt-tolerant sweetpotato crop in order to use the marginal and saline lands. “Xushu 32” and “Xushu 22” are two sweetpotato cultivars which are largely planted in China. “Xushu 32” was derived from parental materials “Xushu55-2” and “Beniazuma” and displays good resistance to black rot and strong storage ability. And “Xushu 22” was generated from parental materials “Yushu 7” and “Sushu 7” and possesses the characteristics of high starch content, high yield and wide adaptability. In our previous studies, we found that “Xushu 22” exhibited better capacity controlling Na^+^, Cl^–^, K^+^ and Mg^2+^ homeostasis than “Xushu 32,” thus displayed good resistance to salt stress (Yu et al., 2016). Then, we observed the transcriptome and proteome in these two cultivars (Meng et al., 2020). In addition, candidate miRNAs related to the storage root development of sweetpotato were identified in salt-tolerant cultivar “Xushu 22” (Tang et al., 2020; Sun L. et al., 2022). Recently, we identified hundreds miRNAs in salt-sensitive cultivar “Xushu 32” by high-throughput deep sequencing and demonstrated that miRNAs play an important role in sweetpotato under salt stress adaptation (Yang et al., 2020). However, the miRNAs in different genotypes of sweetpotato with different salt sensitivity have not been comparatively analyzed.

Combined with previously reported miRNA data from the salt-sensitive cultivar “Xushu 32,” we performed a comparative analysis of the miRNAs between the two sweetpotato cultivars after we identified the miRNAs from the salt-tolerant “Xushu 22” by high-throughput sequencing. The purpose of this study was to (1) investigate the changes in miRNAs and regulatory patterns that may lead to the differences in the salt tolerance of the two genotypes of sweetpotato under salt stress, (2) improve our understanding of the genetic regulation mechanisms of salt stress response in sweetpotato and (3) provide valuable information for further studies on the potential miRNAs that improve the resistance of sweetpotato.

## Materials and Methods

### Experimental Materials and Salt Treatment

The two sweetpotato cultivars “Xushu 22” (salt-tolerant genotype) and “Xushu 32” (salt-sensitive genotype) used as experimental materials were supplied by Jiangsu Xuzhou Sweetpotato Research Centre, Xuzhou, China. The plant culture and salt treatment methods are similar to that in our previous study (Yang et al., 2020). Briefly, sweetpotato shoots were grown in 1/2 Hogeland solution for 10 days to generate four or five functional leaves, and then the plants with uniform phenotype were subjected to 150 mM NaCl treatment for 2 days. Plants without salt treatment were used as the control group. Sweetpotato roots and leaves were separately harvested, frozen by liquid nitrogen and stored at −80°C for subsequent experiments.

### Small RNA Library Construction and Sequencing

RNA was extracted from XLC (NaCl-untreated “Xushu 22” leaves, as a control group), XLN (NaCl-treated “Xushu 22” leaves, as a treatment group), XRC (NaCl-untreated “Xushu 22” roots as a control group) and XRN (NaCl-treated “Xushu 22” roots, as a treatment group) according to the manufacturer’s instructions of TRIzol reagent (Invitrogen, CA, United States). A total of 12 RNA libraries from the four groups with three independent biological replicates were constructed and sequenced by Illumina HiSeq 2000/2500 as previously described (Yang et al., 2020). Briefly, the 5′ and 3′ adapter was successively ligated to the RNA fragments with T4 RNA ligase. The resulting small RNA were reverse transcribed to cDNA and then subjected to PCR with 11 cycles using the adaptor primers. The PCR product fragments with the size of 140–160 bp were isolated on 6% polyacrylamide Tris-borate-EDTA gel and then sequenced.

### MiRNA Identification in Sweetpotato by High Throughput Sequencing

MiRNAs were identified in “Xushu 22” cultivar similarly as in “Xushu 32” (Yang et al., 2020). In brief, original data was first processed and analyzed by ACGT101-miR v3.5 software (LC Sciences, Huston, TX, United States). Small RNAs were compared with known non-redundant plant mature miRNAs in miRBase 21.0 (one mismatch is allowed in the alignment) using Bowtie software, and the unique sequences mapped to specific species mature miRNAs were considered miRNAs. The matching sequences are considered to be known miRNAs, and did not match sequences using Mfold software^1^ predicted its structure and then obtained novel miRNA according to previous criteria (Meyers et al., 2008; Li et al., 2013). The criteria for secondary structure prediction were: (a) number of nucleotides in one bulge in stem ≤ 12, (b) number of base pairs in the stem region of the predicted hairpin ≥ 16, (c) cutoff of free energy kCal/mol ≤ 15, (d) length of hairpin (up and down stems + terminal loop ≥ 50), (e) length of hairpin loop ≤ 200, (f) number of nucleotides in one bulge in mature region ≤ 4, (g) number of biased errors in one bulge in mature region ≤ 2, (h) number of biased bulges in mature region ≤ 2, (i) number of errors in mature region ≤ 4, (j) number of base pairs in the mature region of the predicted hairpin ≥ 12, (k) percent of mature in stem ≥ 80.

### Construction and Sequencing of Transcriptome and Degradome Libraries

Total RNA from the four samples (XLC, XLN, XRC and XRN) of sweetpotato cultivar “Xushu 22” was processed for library construction, transcriptome sequencing and assembly according to a previous method (Xie et al., 2018; Yang et al., 2020). Briefly, approximately 5 μg of total RNA was subjected to isolate Poly (A) mRNA with poly-T oligo attached magnetic beads (Invitrogen). Then the cleaved RNA fragment ligated 3′ and 5′ adapter. Based on the adapter sequence, a reverse transcription was used to create cDNA constructs. The average insert size for the paired-end libraries was 400 bp (+ 50 bp). And then it sequenced on an Illumina Hiseq 2500 platform. To investigate the target genes of the miRNAs, the mixed root and leaf samples from the NaCl-untreated group (DXC) and NaCl-treated group (DXN) were used to construct the degradome library for sequencing as previously described (Addo-Quaye et al., 2008, 2009). Briefly, approximately 150 ng of poly(A) + RNA was used as input RNA and annealing with Biotinylated Random Primers. A reverse transcription followed by PCR was used to create cDNA constructs. Then libraries were sequenced using the 5′ adapter, resulting in the sequencing of the first 36 nucleotides of the inserts that represented the 5′ ends of the original RNA.

### Verification of MiRNA Expression by Stem-Loop Quantitative Real-Time Polymerase Chain Reaction

We carried out stem-loop quantitative real-time polymerase chain reaction (qRT-PCR) to confirm the expression of miRNAs and verify the results of high-throughput sequencing. About 1 μg DNA-free total RNA was hybridized with miRNA-specific stem-loop RT primers, and the hybridized molecules were reverse-transcribed into cDNAs by using the PrimeScript™ RT Reagent Kit (Takara, Dalian, China). And then qRT-PCR was performed on the ABI system (ABI, United States) as described previously (Lu et al., 2019). The expression of miRNA targets were also checked as same as the previous methods (Yang et al., 2020). The ADP- ribosylation factor gene was used as internal reference. The reverse transcription and qRT-PCR primer are listed in Supplementary Table 1. Each experiment was repeated at least three times.

### Analysis of Differentially Expressed MiRNAs and GO Enrichment on DEGs of Target Genes in Sweetpotato

The differentially expressed miRNAs were determined based on the relative expression abundance of each miRNA, and *p*-value ≤ 0.05 and |log_2_(fold change)| ≥ 1 were the thresholds. Genes that are significantly differently expressed meet the following criterion: |log_2_(fold change)| ≥ 1 and *p*-value ≤ 0.05; genes that have extremely significant difference expression meet the following criterion: |log_2_ (fold change)| ≥ 1 and *p*-value ≤ 0.01.

GO enrichment analysis provides all GO terms that significantly enriched in target genes of DEMs. Firstly, all target genes were mapped to GO terms in the Gene Ontology database^2^, gene numbers were calculated for every term, significantly enriched GO terms in target genes were defined by hypergeometric test. Then *p*-value was calculated (*p*-value ≤ 0.05 as the threshold). GO terms meeting this condition were defined as significantly enriched GO terms.

## Results

### Analysis of Transcriptome and Small RNA Sequencing on “Xushu 22” Sweetpotato Cultivar

Transcriptome sequencing produced about 462.31 million valid reads from the four libraries of “Xushu 22” samples (XRC, XLC, XRN and XLN) with an average of 115.8 million valid reads per sample reading (Supplementary Table 2). The Q30 scores of all samples ranged from 93.11% to 94.45% with an average of 93.64%; thus, the quality of the sequence data is reliable. And these transcriptome data were used as the sweetpotato reference sequence.

**FIGURE 1:**
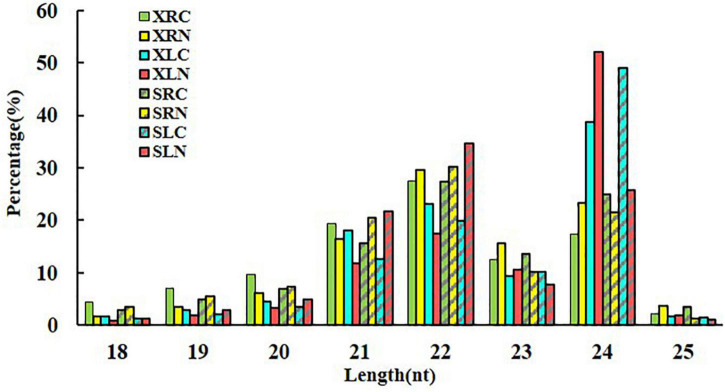
Length distribution of small RNAs in two sweetpotato cultivars. XRC: “Xushu 22” root without NaCl treatment as control; XRN: “Xushu 22” root with NaCl treatment; XLC: “Xushu 22” leaf without NaCl treatment as control; XLN: “Xushu 22” leaf with NaCl treatment; SRC: “Xushu 32” root without NaCl treatment as control; SRN: “Xushu 32” root with NaCl treatment; SLC: “Xushu 32” leaf without NaCl treatment as control; SLN: “Xushu 32” leaf with NaCl treatment.

Small RNA sequencing has been performed in sweetpotato cultivar “Xushu 32” in our previous study (Yang et al., 2020). In the present study, another 12 small RNA libraries constructed from cultivar “Xushu 32” was sequenced to perform a comparative analysis of salt responsive miRNAs in the two different sweetpotato genotypes with different salt stress sensitivity. A total of more than 211.44 million raw reads were obtained in “Xushu 22” RNA libraries after removing low-quality, repetitive sequences and substandard sequences (less than 18 nucleotides or more than 25 nucleotides). About 90 million valid reads were obtained in total, and each sample had about 7.5 million valid reads (Supplementary Table 3). The original data were deposited in the National Centre for Biotechnology Information database (Nos. from SRR11095920 to SRR11095931). The length distribution of small RNA in “Xushu 22” libraries is concentrated at 21, 22 and 24 nt, which is consistent with the length distribution in “Xushu 32” (Yang et al., 2020). However, small RNA with a length of 22 nt was observed in XRN and SLN, and small RNA with a length of 24 nt was mostly in XLN and SLC (Figure 1).

### Identification of MiRNA in “Xushu 22”

**FIGURE 2:**
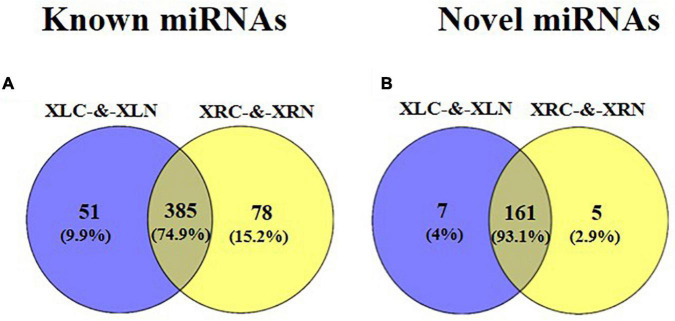
Distribution of known and novel miRNAs in roots and leaves of “Xushu 22.” **(A)** Known miRNAs in roots and leaves; **(B)** Novel miRNAs in roots and leaves.

A total of 687 miRNAs were identified in “Xushu 22” (Figure 2 and Supplementary Tables 4, 5), of which 514 are known miRNAs and 173 are novel miRNAs. Among the 514 known miRNAs, 51 and 78 miRNAs were specially expressed in “Xushu 22” leaves and roots, respectively, and 385 were co-expressed in roots and leaves (Figure 2A). Among the 173 novel miRNAs, five and seven were specifically expressed in roots and leaves, respectively, and 161 were co-expressed in roots and leaves (Figure 2B).

### Comparative Analysis of MiRNA Distribution Between “Xushu 22” and “Xushu 32”

**FIGURE 3:**
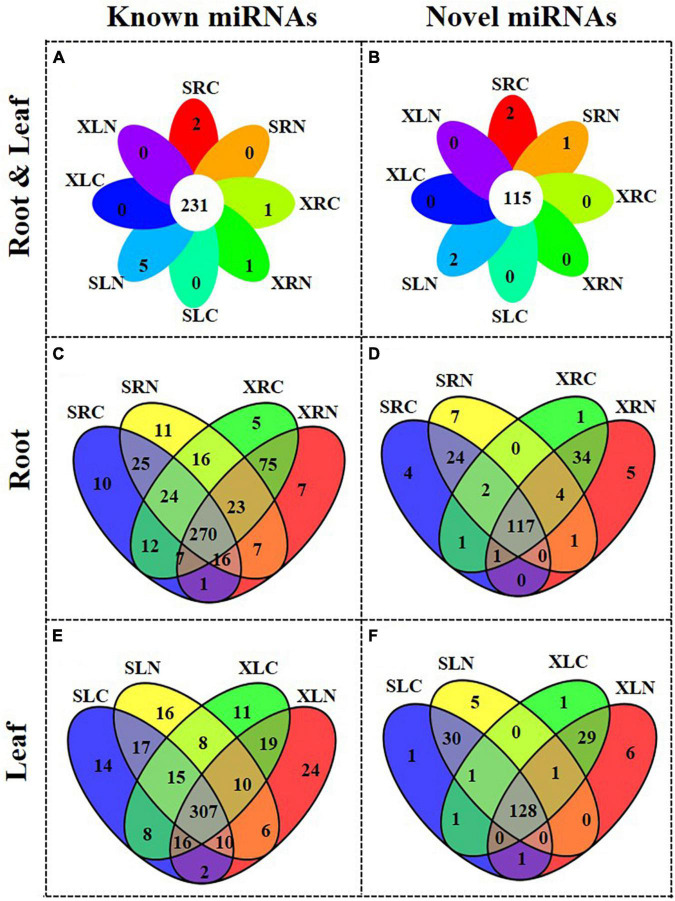
Distribution of known and novel miRNAs in roots and leaves of two sweetpotato cultivars “Xushu 22” and “Xushu 32.” **(A)** Known miRNAs in roots and leaves; **(B)** Novel miRNAs in roots and leaves; **(C)** Known miRNAs in roots; **(D)** Novel miRNAs in roots; **(E)** Known miRNAs in leaves; **(F)** Novel miRNAs in leaves.

Figures 3A,B show the overview of the miRNA distribution in “Xushu 22” (including XLC, XLN, XRC and XRN) and “Xushu 32” (SLC, SLC, SRC and SRN). A total of 231 known miRNAs and 115 novel miRNAs were co-expressed in each of the libraries of “Xushu 22” and “Xushu 32.” Two known miRNAs (ptc-MIR7836-p5_2ss4TG19GA and Gma-MIR9740-p3_2ss12CA21AT) and two novel miRNAs (PC-5p-1546858_11 and PC-3p-1239497_15) were only expressed in SRC (Figures 3A,B and Supplementary Table 6); one known miRNA (aly-MIR169k-p5_2ss13AG18AT) and another known miRNA (gma-MIR1524-p5_2ss5CA19GA) were only expressed in XRC and XRN, respectively (Figure 3A and Supplementary Table 6); five known miRNAs (zma-MIR395c-p3_1ss6TC, bna-MIR166b-p3_1ss20TC, ptc-miR167f-5p_L-2R + 1, osa-MIR1871-p3_2ss18GA21TG and ath-MIR5645e-p5_2ss17TG18GT) and two novel miRNAs (2 PC-3p-454675_50 and PC-5p-150993_155) were only expressed in SLN (Figures 3A,B); one novel miRNA (PC-3p-862361_24) was only expressed in SRN (Figure 3B and Supplementary Table 6).

In sweetpotato roots, 270 known miRNAs and 117 novel miRNAs were co-expressed in “Xushu 32” and “Xushu 22” (Figures 3C,D and Supplementary Tables 4–6). Forty-six known miRNAs and 35 novel miRNAs were expressed in “Xushu 32” root but not in “Xushu 22” root. Among them, 10 known miRNAs (far-miR159_L + 2_1ss22TC, gma-MIR159a-p5_2ss7TA19CA, nta-MIR166d-p5_1ss13TA, mdm-MIR482b-p3_3ss9CA21TC22TA, etc.) and four novel miRNAs were only expressed in SRC, whereas 11 known miRNAs (stu-miR160a-5p_R + 4, stu-miR169a-5p_R + 2_1ss1TA, stu-miR396-5p_2ss20TC21TC, gma-miR159a-3p_L + 1_1ss21TC, etc.) and seven novel miRNAs were only expressed in SRN. Eighty-seven known miRNAs and 40 novel miRNAs are only expressed in “Xushu 22” root but not in “Xushu 32” root. Among them, five known miRNAs and one novel miRNA are only expressed in XRC, and seven known miRNAs and five novel miRNAs were only expressed in XRN.

In sweetpotato leaves, 307 known miRNAs and 128 novel miRNAs were co-expressed in “Xushu 32” and “Xushu 22” (Figures 3E,F and Supplementary Tables 4–6). 47 known miRNAs and 36 novel miRNAs were only expressed in “Xushu 32” leaves but not in “Xushu 22.” Among them, 14 known miRNAs and one novel miRNA were only expressed in SLC, and 16 known miRNAs and five novel miRNAs were only expressed in SLN. Fifty-four known miRNAs and 36 novel miRNAs were only expressed in “Xushu 22” leaves but not in “Xushu 32.” Among them, 11 known miRNAs and one novel miRNA were expressed only in XLC, and 24 known miRNAs and six novel miRNAs were only expressed in XLN (Figures 3E,F).

### Comparative Analysis of the MiRNA Expression Between “Xushu 22” and “Xushu 32” Genotypes

We analyzed the miRNA expression of the two genotypes to better understand the different salt tolerance capabilities of the two sweetpotato cultivars. All the expression levels of known and novel miRNAs in “Xushu 22” and “Xushu 32” were normalized, and the miRNAs from the roots and leaves of the two genotypes under untreated and salt-treated conditions were compared (i.e., XRC VS SRC, XRN VS SRN, XLC VS SLC and XLN VS SLN; Supplementary Table 6).

**FIGURE 4:**
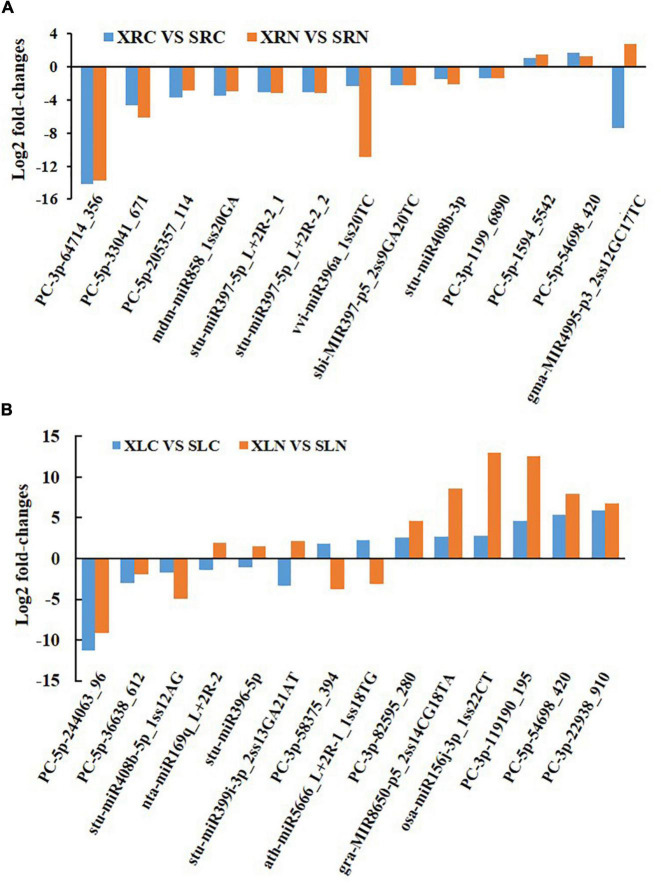
Comparative analysis of miRNA expression between two different genotypes under NaCl treatment or untreated condition. **(A)** Differently expressed miRNAs in roots. **(B)** Differently expressed miRNAs in leaves.

Figure 4A shows the comparative analysis of miRNA expression in the roots between two different genotypes under NaCl treatment and untreated conditions. Two miRNAs (PC-5p-54698_420 and PC-5p-1594_5542) were significantly more highly expressed (log_2_(fold change) ≥ 1 and *p*-value ≤ 0.05) in NaCl-treated and untreated “Xushu 22” than in “Xushu 32,” but 10 miRNAs (stu-miR397-5p_L + 2R-2_1, stu-miR397-5p_L + 2R-2_2, stu-miR408b-3p, vvi-miR396a_1ss20TC, sbi-MIR397-p5_2ss9GA20TC, mdm-miR858_1ss20GA, PC-3p-1199_6890, PC-5p-33041_671, PC-3p -64714_356 and PC-5p-205357_114) were significantly more lowly expressed in “Xushu 22” than in “Xushu 32.” Interestingly, one miRNA (gma-MIR4995-p3_2ss12GC17TC) was substantially lowly expressed in “Xushu 22” in salt-untreated roots but highly expressed in “Xushu 22” salt-treated roots.

Figure 4B shows the comparative analysis of miRNA expression in the leaves between two different genotypes under NaCl-treated or untreated conditions. Six miRNAs (osa-miR156j-3p_1ss22CT, gra-MIR8650-p5_2ss14CG18TA, PC-5p-54698_420, PC-3p-119190_195, PC-3p-22938_910 and PC -3p-82595_280) were significantly more highly expressed in NaCl-treated and untreated “Xushu 22” than in “Xushu 32,” but three miRNAs (stu-miR408b-5p_1ss12AG, PC-5p-244063_96 and PC-5p-36638_612) were significantly more lowly expressed in “Xushu 22” than in “Xushu 32.” Three miRNAs (stu-miR396-5p, stu-miR399i-3p_2ss13GA21AT and nta-miR169q_L + 2R-2) were remarkably lowly expressed in the salt-untreated leaves of “Xushu 22” but considerably highly expressed in salt-treated leaves. By contrast, two miRNAs (ath-miR5666_L + 2R-1_1ss18TG and PC-3p-58375_394) were remarkably highly expressed in “Xushu 22” salt-untreated leaves but substantially lowly expressed in “Xushu 22” salt-treated leaves.

### Comparative Analysis of Salt-Responsive MiRNAs in “Xushu 22” and “Xushu 32”

**FIGURE 5:**
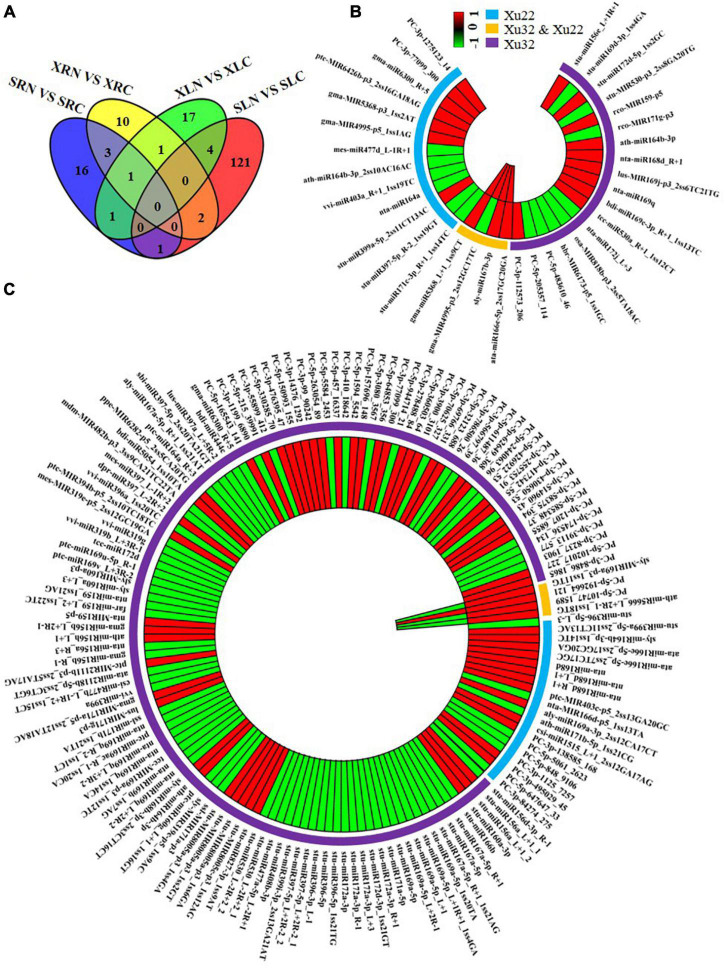
Salt-responsive miRNAs in leaves and roots. **(A)** Salt-responsive miRNAs in roots and leaves; **(B)** Salt-responsive miRNAs in roots; **(C)** Salt-responsive miRNAs in leaves. Each bar in panels **(B,C)** represents a miRNA. Red bars represent miRNAs (salt/control) with log_2_ (fold change) ≥ 1; green bars represent miRNAs (salt/control) with log_2_ (fold change) ≤ –1; the blue outer ring represents miRNAs with significant differential expression in “Xushu 22”; the yellow outer ring represents miRNAs with significant differential expression in “Xushu 32” and “Xushu 22”; the purple outer ring only represents miRNA with significant differential expression in “Xushu 32.” The first and second layer from the center of panels **(B,C)** indicates “Xushu 22” and “Xushu 32,” respectively. Significant difference between salt-stressed sweetpotato and control sweetpotato is indicated by *p*-value ≤ 0.05.

We compared the miRNA expression levels between the salt-treated group and their corresponding control group for each genotype to identify the miRNAs that respond to salt stress in sweetpotato. A total of 39 miRNAs (28 known and 11 novel miRNAs) were differentially and substantially expressed in “Xushu 22” under salt stress (Figure 5A and Supplementary Table 6). In “Xushu 22” leaves, 14 miRNAs (four novel and 10 known miRNAs) were up-regulated by salt stress, and 10 miRNAs (five novel and five known miRNAs) were down-regulated by salt stress. In “Xushu 22” roots, 11 miRNAs (two novel and nine known miRNAs) were up-regulated by salt stress, and six miRNAs (six known miRNAs) were down-regulated by salt stress. Only two miRNAs co-expressed in the roots and leaves of “Xushu 22” (stu-miR399a-5p_2ss11CT13AC and ata-miR166e-5p_2ss17GC20GA) were up-regulated by salt stress (Supplementary Table 6). In the four comparison groups (SRN VS SRC, XRN VS XRC, XLN VS XLC and SLN VS SLC), a total of 177 miRNAs were substantially and differentially expressed under salt stress, of which 16, 10, 17 and 121 miRNAs were only substantially and differentially expressed in SRN VS SRC, XRN VS XRC, XLN VS XLC and SLN VS SLC, respectively (Figure 5A and Supplementary Table 6).

Eighteen miRNAs were significantly regulated by salt stress only in the roots of “Xushu 32” (Figures 5A,B). Among which 10 miRNAs (stu-miR156e_L + 1R + 1, stu-miR172d-5p_1ss2GC, rco-miR159-p5, ath-miR164b-3p, nta-miR168d_R + 1, lus-miR169j-p3_2ss6TC21TG, nta-miR169q, bdi-miR169c-3p_R + 1_1ss13TC, tcc-miR530a_R + 1_1ss12CT and PC-3p-112573_206) were up-regulated by salt stress, and eight miRNAs (stu-miR169d-3p_1ss4GA, stu-miR530-p3_2ss8GA20TG, rco-miR171g-p3, nta-miR172j_L + 3, osa-miR818b-p3_2ss5TA18AC, hbr-miR6173-p5_1ss1GC, PC-5p-483610_46 and PC-5p-205357_114) were down-regulated (Figure 5B). Thirteen miRNAs were remarkably regulated by salt stress only in the roots of “Xushu 22,” of which seven miRNAs (stu-miR399a-5p_2ss11CT13AC, gma-MIR4995-p5_1ss1AG, gma-MIR5368-p3_1ss2AT, ptc-MIR6426b-p3_2ss16GA18AG, gma-miR6300_R + 5, PC-3p-99 and 3p-1275123_14) were up-regulated by salt stress and six miRNAs (stu-miR171c-3p_R + 1_1ss14TC, stu-miR397-5p_R-2_1ss19GT, nta-miR164a, vvi-miR403a_R + 1_1ss19TC, ath-miR164b-3p_2ss10AC16AC and mes-miR477d_L) were down-regulated (Figure 5B). Four miRNAs were substantially upregulated by salt stress in “Xushu 32” and “Xushu 22” roots (Figure 5A), of which three miRNAs (ata-miR166e-5p_2ss17GC20GA, sly-miR167b-3p and gma-miR5368_L + 1_1ss9CT) were up-regulated and one miRNA (gma-miR4995-p3_2ss12GC17TC) was up-regulated in “Xushu 22” but down-regulated in “Xushu 32” (Figure 5B).

A total of 124 miRNAs were significantly regulated by salt stress only in the leaves of “Xushu 32” (Figures 5A,C), of which 47 miRNAs (stu-MIR8005a-p3_1ss4GA, ath-miR164b-3p, sly-miR168b-3p_2ss3CT16CT, lus-MIR171g-p3, etc.) were up-regulated by salt stress and 77 miRNAs (stu-miR172a-3p, stu-miR396-5p, stu-miR397-5p_L + 2R-2_1, stu-miR408b-3p, etc.) were down-regulated (Figure 5C). Twenty miRNAs were remarkably regulated by salt stress only in the leaves of “Xushu 22”; (Figure 5A), of which 13 miRNAs (stu-miR399a-5p_2ss11CT13AC, sly-miR164b-3p_1ss14TC, ata-miR166e-5p_2ss7TC17GC, etc.) were up-regulated by salt stress and seven miRNAs (stu-miR396-5p_L-3, nta-miR166d-p5_1ss13TA, ath-miR171b-5p_1ss21CG, PC-5p-5061_2623, PC-5p-848_9106, PC-3p-1125_7257 and PC-3p-84274_275) were down-regulated. Four miRNAs were substantially regulated by salt stress in “Xushu 32” and “Xushu 22” leaves, of which one miRNA (PC-5p-192664_121) was up-regulated in “Xushu 32” and “Xushu 22,” and three miRNAs (sly-miR169a-p3_1ss11TG, ath-miR5666_L + 2R-1_1ss18TG and PC-5p-10747_1589) were down-regulated in “Xushu 22” but up-regulated in “Xushu 32” (Figure 5C).

### Identification of MiRNA Target Genes in Different Genotypes

We used Target Finder and degradome sequencing to predict and identify the target genes of sweetpotato miRNAs (Table 1). Although 294 miRNAs were substantially regulated by salt stress or differently expressed between the two genotypes, 1,668 target genes (991 and 677 target genes from Target Finder and degradome sequencing, respectively.) were predicted and identified only for 230 miRNAs (Supplementary Table 7). Among these miRNAs, 56 target transcription factors related to growth and stress, such as *MYB*, *NAC*, *WRKY*, *GRF*, *SBP*, *AP2/ERF, NFYA*, *TCP*, *SPL*, *LBD*, *DREB*, and *DOF*. Some miRNAs target resistance protein-related genes. For example, bdi-MIR7716-p5_2ss12TC19AT targets *HSP23*, *HSP18.5-C*, *HSP83A* and *HSP90*; stu-miR162a-3p and stu-miR396-5p_L-3 target *HSP70*; and zma-MIR2275b-p5_2ss3CT17AT targets *HSP70-17*. Some miRNAs target antioxidant-related genes. For instance, PC-5p-205357_114 targets *CAT4*, osa-MIR818b-p3_2ss5TA18AC targets *CAT2*, ptc-MIR6426b-p5_2ss13GA19GA targets *PEX22*, stu-miR172d-5p_1ss2GC targets *FER2* and ptc-MIR169k-p5_1ss11GT targets *GSH1*. Some miRNAs target kinases (calcium-dependent protein kinases) related to salt stress signal transduction. For example, nta-miR169q_L + 2R-2 targets *CPK32*, and zma-MIR2275b-p5_2ss3CT17AT targets *CPK18* and *CPK13*.

**TABLE 1:** Summary of the degradome sequencing data in DXN and DXC.

Sample	DXN		DXC	
		
	Number	Ratio	Number	Ratio
Raw reads	17,719,993	/	17,031,395	/
Reads < 15 nt after removing 3 adaptor	66,486	0.38%	64,512	0.38%
Mappable reads	17,653,507	99.62%	16,966,883	99.62%
Unique raw reads	9,850,392	/	6,533,843	/
Unique reads < 15 nt after removing 3 adaptor	45,049	0.46%	38,643	0.59%
Unique mappable reads	9,805,343	99.54%	6,495,200	99.41%
Transcript mapped reads	7,922,669	44.71%	10,353,414	60.79%
Unique transcript mapped reads	2,370,127	24.06%	2,450,707	37.51%
Number of input transcript	27,712	/	27,712	/
Number of covered transcript	24,868	89.74%	25,033	90.33%

**FIGURE 6:**
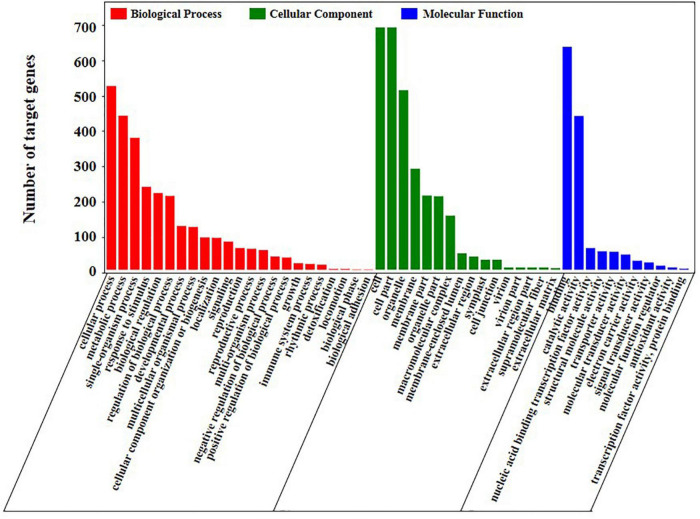
GO analysis of the target genes of differentially expressed miRNAs in genotype and salt stress response.

We performed Gene Ontology (GO) enrichment analysis of the target genes to further understand the function of these DEMs. A total of 1,688 potential miRNA targets were classified according to three basic functions, namely, biological processes, cellular components and molecular functions (Figure 6 and Supplementary Table 8). The result indicated that 247 target genes were significantly enriched on the GO terms (*p*-value ≤ 0.05) that related to stress response [GO:0009628 (response to abiotic stimulus), GO:0050896 (response to stimulus), GO:0006950 (response to stress) and GO:0006970 (response to osmotic stress)], oxidoreductase activity (GO:0016703, GO:0016701 and GO:1901700) and potassium ion transport (GO:0010107, GO:0005267 and GO:0015079).

### QRT-PCR Validation of MiRNA Expression

**FIGURE 7:**
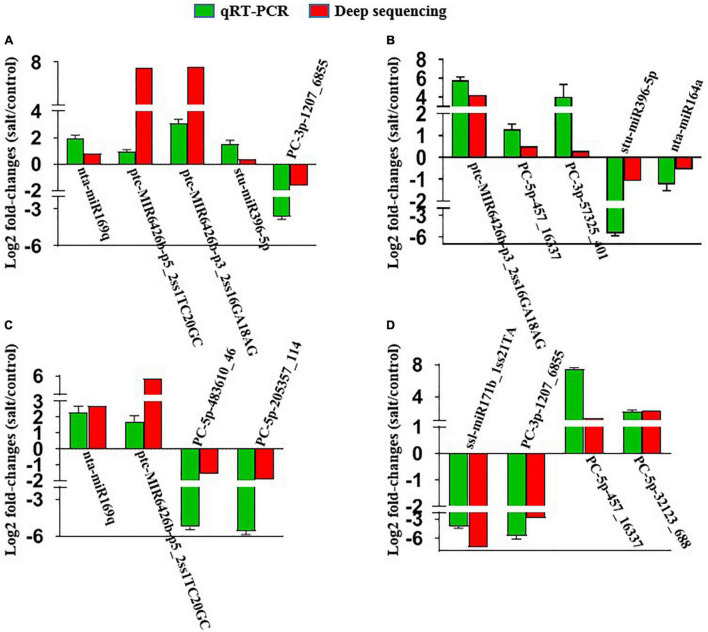
QRT-PCR validation of randomly selected salt-responsive miRNAs in the leaves and roots of two sweetpotato cultivars. **(A)** MiRNAs in “Xushu 22” roots; **(B)** miRNAs in “Xushu 22” leaves; **(C)** miRNAs in “Xushu 32” roots; **(D)** miRNAs in “Xushu 32” leaves. Each bar represents a miRNA with log_2_ fold change (2^–ΔΔ*Ct*^) in expression. Data are displayed as averages ± SD (n = 3 PCR replicates).

We checked the expression levels of five miRNAs in “Xushu 22” leaves and roots by performing qRT-PCR experiments to confirm the results of deep sequencing and the real expression of miRNAs. The expression levels of another four miRNAs from “Xushu 32” were also verified in this study. We also validated other miRNAs in the previous study (Yang et al., 2020). Figure 7 displays the fold changes of the expression level in the salt treatment group compared with the control group. The results showed that the expression levels from qRT-PCR experiments were consistent with those from the deep sequencing results (Figure 7).

The results of qRT-PCR showed that four miRNAs (nta-miR169q, ptc-miR6426b-p5_2ss1TC20GC, ptc-miR6426b-p3_2ss16GA18AG and stu-miR396-5p) were up-regulated and PC-3p-1207_6855 was down-regulated in the roots of “Xushu 22” under salt stress (Figure 7A), and three miRNAs (ptc-miR6426b-p3_2ss16GA18AG, PC-5p-457_16337 and PC-3p-57325_401) were up-regulated and two miRNAs (stu-miR396-5p and nta-miR164a) were down-regulated in the leaves of “Xushu 22” under salt stress (Figure 7B). Nta-miR169q and ptc-miR6426b-p5_2ss1TC20GC were up-regulated whereas PC-5p-483610_46 and PC-5p-205357_114 were down-regulated in “Xushu 32” roots under salt stress (Figure 7C). Ssl-miR171b_1ss21TA and PC-3p-1207_6855 were down-regulated whereas PC-5p-457_16337 and PC-5p-32123_688 were up-regulated in “Xushu 32” leaves under salt stress (Figure 7D). These results were positively correlated with the deep sequencing results.

## Discussion

Soil salinization is the main factor that restricts the growth and agricultural productivity of plants worldwide. Sweetpotato is an important food crop, but its production is limited by the increase in saline land. Therefore, extensive efforts are needed to discover the genetic factors and mechanisms to enhance the salt tolerance of crops. In this study, the miRNA expression profiles of salt-sensitive and salt-tolerant sweetpotato genotypes under salt treatment and non-treatment conditions were analyzed to provide useful information for understanding the role of miRNAs and improving the agronomic traits of sweetpotato under salinity stress.

Plant roots are in direct contact with the soil environment and are the primary organs for sensing external salt stress signals. The propagation mode of sweetpotato in agriculture farming is asexual reproduction using cottage method. Adventitious roots at the early stage of the seedlings are susceptible to salt stress. MiRNAs that target transcription factors related to root development in response to environmental stimuli play an important role in regulating root growth or lateral root formation and therefore adapt root structure to the soil environment (Khan et al., 2011). For example, the miRNA164-mediated cleavage of *NAC1* affects lateral root development in maize and *Arabidopsis*, and the overexpression of Z*mNAC1* and *AtNAC1* in *Arabidopsis* increases the number of lateral roots (Guo et al., 2005; Lu et al., 2017). Additionally, miR164 negatively regulates its target gene *NAC* and also plays an important role in plant response to salt stress. Transgenic plants that overexpress transcription factors from the NAC family, such as *Arabidopsis*, chickpea, rice, wheat, cotton and tomato, have displayed improved salt tolerance compared with wild-type plants (Nakashima et al., 2007; Peng et al., 2009; Yokotani et al., 2009; Xue et al., 2011; Han et al., 2012; Liu et al., 2014). In the present study, nta-miR164a and nta-miR164a_R + 1 were down-regulated by NaCl treatment in “Xushu 32” and “Xushu 22” sweetpotatoes. Our degradome sequencing results indicated that the miRNAs of these two sweetpotato cultivars target the NAC transcription factor; therefore, the miRNAs of sweetpotato that belong to the miR164 family may negatively affect salt stress response by regulating *NAC* transcription factors. Meanwhile, nta-miR164a and nta-miR164a_R + 1 were lowly expressed in the roots of salt-tolerant cultivar “Xushu 22” comparing with those in the roots of salt-sensitive cultivar “Xushu 32” under NaCl treatment and untreated conditions. Degradome sequencing showed that nta-miR164a_R + 1 targeted comp23998_c0 (*NAC021*) (Supplementary Table 8). This result implied that miR164 family members in sweetpotato may be involved in lateral root development and the difference in the salt tolerance between these two sweetpotato genotypes and need to be further studied.

Nta-miR169q, which targets *NF-YA2/9*, was induced by salt stress in “Xushu 32” and “Xushu 22” roots. In *Arabidopsis*, the miR169-*NFYA2*/*10* module regulates the growth of main roots and the occurrence of lateral roots, and transgenic plants overexpressing wheat *NF-YA10* and *GmNF-YA3* show sensitivity to high salt stress (Ni et al., 2013; Sorin et al., 2014; Ma et al., 2015). The expression of sweetpotato nta-miR169q induced by salt stress may negatively regulate the expression of the target gene *NF-YA*; therefore, this miRNA regulates the growth of sweetpotato roots and improves the salt tolerance of sweetpotatoes.

A total of 31 miR156 family members have been identified in sweetpotatoes, of which 20 miRNAs target plant-specific transcription factors in the *SPL* gene family. MiR156a and miR156c are substantially induced in *Arabidopsis* under salt and drought stress, and 35S:MIM156 (targets mimicry) is extremely sensitive to salt and drought stress (Cui et al., 2014). The overexpression of miR156d in alfalfa increases tolerance to drought and salt stress by silencing the target gene *SPL13* (Cui et al., 2014; Arshad et al., 2017a,b). Under NaCl-untreated conditions, the expression levels of 22 miR156 family members in “Xushu 22” roots were higher than those in “Xushu 32” roots. What’s more, about half (15 in 31) of the miR156 family members were expressed higher in “Xushu 22” roots than in “Xushu 32” roots under salt treatment condition. This result suggested that miR156 may play an important role related to the difference in salt sensitivity of these two sweetpotato genotypes.

Twenty members of miR6426 family were identified, and only two miRNAs (ptc-miR6426b-P3_2SS16GA18AG and ptc-miR6426b-p5_2SS1TC20GC) were induced by salt stress and lowly expressed in “Xushu 32.” All 20 miR6426 family members were expressed in “Xushu 22” roots, and 14 of which were induced by salt stress. Nineteen miR6426 family members were expressed in “Xushu 22” leaves, and 18 of them were induced by salt stress. MiR6426 promotes *Citrus Sinensis* tolerance to magnesium-deficiency stress and participates in tomato drought stress response (Candar-Cakir et al., 2016; Ma et al., 2016). Our study found that miR6426 was expressed in salt-tolerant sweetpotato “Xushu 22” but not in “Xushu 32,” and ptc-miR6426b-p3_2ss16GA18AG was remarkably up-regulated under salt stress as confirmed by qRT-PCR. Degradome results indicated that miR6426 targets stress-related calmodulin kinase and transcription factors (*HAT*, *bHLH*, *RAP2*, *MYB* and *ERF*). Thus, we inferred that the *miR6426* gene family might be involved in the difference of the salt tolerance between these two sweetpotato genotypes.

Tu-miR397-5p_L + 2R-2_1 and stu-miR397-5p_L + 2R-2_2 in the miR397 family were remarkably lowly expressed in “Xushu 22” roots than in “Xushu 32” roots under salt-treated and untreated conditions. Stu-miR397-5p_R-2_1ss19GT was down-regulated in “Xushu 22” and “Xushu 32” roots under salt stress. The predicted target gene of tu-miR397-5p_L + 2R-2_1, stu-miR397-5p_L + 2R-2_2 and stu-miR397-5p_R-2_1ss19GT was *LAC7*. In tomato and corn roots, high NaCl concentrations can increase the expression level of lignin synthesis genes (Wei et al., 2000). Laccase plays an important role in root development and salt stress adaptation in *Arabidopsis* and *Poncirus trifoliate* (Cai et al., 2006; Turlapati et al., 2011). In sweetpotatoes, the cleavage of the laccase gene may be reduced to reduce the expression of miR397 and promote root growth and tolerance to salt stress. In addition to some known miRNAs, some novel miRNAs that respond to salt stress in roots were also found. For example, PC-5p-483610_46 was remarkably down-regulated by salt stress in “Xushu 32” roots but not expressed in “Xushu 22” roots. Degradome sequencing identified that PC-5p-483610_46 targets comp19781_c0. The function of the target genes of these novel miRNAs needs to be further studied in the future.

Leaves are more sensitive to salt stress than plant roots during osmotic stress because salt accumulates rapidly in leaves (Redondo-Gómez et al., 2007). The toxicity mechanism of salt ions is to accelerate the ageing of tissues and organs, which will cause the mature leaves to lose the ability to dilute the salt; therefore, salt reduces a plant’s ability to detoxify (Redondo-Gómez et al., 2007). Our study found that the expression of stu-miR396-5p and stu-miR396-3p_L-1 was remarkably lower in “Xushu 22” leaves than that in “Xushu 32” leaves under NaCl-untreated condition, but the expression of stu-miR396-5p and stu-miR396-5p_1ss21TG was substantially higher in “Xushu 22” leaves than in “Xushu 32” leaves under salt treatment condition. Vvi-miR396a_1ss20TC, stu-miR396-5p and stu-miR396-5p_1ss21TG of the miR396 family were down-regulated in “Xushu 32” leaves under salt stress. Degradome sequencing identified that vvi-miR396a_1ss20TC, stu-miR396-5p and stu-miR396-5p_1ss21TG target (growth-regulating factor (*GRF*) family members, including *GRF2*, *GRF3* and *GRF6*. The miR396-GRF regulatory module plays an important role in plant leaf development and stress response (Hewezi and Baum, 2012). Transgenic *Arabidopsis* that over-expressed miR396 inhibited *GRF* expression, its leaves became smaller and narrower, the number of stomata decreased, and the plant became more drought-tolerant than the wild type (Liu et al., 2009; Mecchia et al., 2012). In tobacco, the overexpression of sp-miR396a-5p increases the tolerance of transgenic plants to salt stress. Compared with wild-type and *ATGRF7* overexpressing lines, *Arabidopsis ATgrf7-1* mutant has increased resistance to salt stress (Kim et al., 2012). These results indicated that miR396 may regulate sweetpotato salt tolerance by suppressing the expression of its target gene *GRF*, and this regulatory module of miR396-GRF may be related to the difference of salt tolerance between “Xushu 22” and “Xushu 32.”

The miR171 family is a conserved miRNA family. In our study, we found that rco-MIR171g-p3 and stu-miR171c-3p_R + 1_1ss14TC were significantly down-regulated by salt stress in “Xushu 32” and “Xushu 22” roots. Ssl-miR171b_1ss21TA and ssl-MIR171a-p3 were significantly down-regulated by salt stress in “Xushu 32” leaves. MiR171, which targets *SCL* of the GRAS family, is down-regulated by salt stress in *S. Alterniflora* leaves and *S. linnaeanum* roots (Zhuang et al., 2014; Qin et al., 2015), affects *Arabidopsis* root and leaf development (Wang et al., 2010) and regulates the salt tolerance of rice (Pauluzzi et al., 2012). Thus, miR171 may also participate in root and leaf development and salt stress response by regulating *SPL* in sweetpotato.

The target gene of miR172 is the *AP2*/*ERF* gene family; *AP2*/*ERF* transcription factors are involved in various biological processes, including drought, high-salt and low-temperature stress responses (Zhuang et al., 2014). Four members of the miR172 family (stu-miR172d-3p_1ss21GT, stu-miR172a-3p_R-1, stu-miR172a-3p and tcc-miR172d) were down-regulated in “Xushu 32” and “Xushu 22” leaves under salt stress. Their down-regulation is consistent with the down-regulation of miR172 in eggplant (*Solanum linnaeanum*) under salt stress. The target genes of miR172 were *AP2* and *RAP2* as identified by degradome sequencing. Leaf yellowing was reduced and salt tolerance was enhanced in tobacco overexpressing *Papaver somniferum AP2* (Mishra et al., 2015), and transgenic *Arabidopsis* overexpressing sweetpotato *RAP2-12* has improved salt tolerance (Li et al., 2019). Thus, the tolerance of sweetpotato to salt stress might be increased by reducing the expression of miR172 and the inhibition of *AP2* and *RAP2* expression.

In “Xushu 32” leaves, most members of the miR169 family that target the NF-YA transcription factor were considerably down-regulated by salt stress. *ATNF-YA2* and *ATNF-YA10* are targeted by miR169 and can regulate the growth of *Arabidopsis* leaves (Zhang et al., 2017). The rosette leaves of *Arabidopsis NF-YA1*/*2*/*3*/*9* overexpression plants became smaller (Siriwardana et al., 2014), and *PtNF-YA9* overexpressed transgenic *Arabidopsis* plants have smaller leaves with reduced biomass, but both plants show strong drought and salt tolerance (Pauluzzi et al., 2012). The overexpression of *NF-YA2*/*7*/*10* leads to plant dwarfing and late senescence and improves the tolerance to abiotic stress (Bailey-Serres and Voesenek, 2008; Leyva-González et al., 2012). We speculate that in sweetpotato leaves, the expression of miR169, which was negatively regulated by salt stress, may result in the accumulation of its target *NF-YA*, controlled leaf growth and stress adaptation.

Interestingly, GO significant enrichment analysis found that 247 target genes were associated with stress response, oxidoreductase activity and potassium ion transport (Supplementary Table 8). For example, *CCD1* significantly enriched on GO:0016702 and GO:0016702. Degradome sequencing indicated PC-5p-175785_133 targeted *CCD1*. CCD1, a novel small calcium-binding protein, positively regulates osmotic and salt tolerance in rice (Jing et al., 2016), and was responsive to drought and salinity stresses in *Brassica oleracea* (Wei et al., 2022). The expression levels of PC-5p-175785_133 in “Xushu 22” leaves was low than that in “Xushu 32” leaves under NaCl-untreated condition. These results suggested that the down-regulation of PC-5p-175785_133 and the increase of its target genes may contribute to the salt tolerance of “Xushu 22.” *PLC4*, *BLH1, CBL10*, *AGO2* and *CRK2* were significantly enriched on stress related GO terms (GO:0050896, GO:0009628, GO:0050896, GO:0006950 and GO:0006970). Degradome sequencing indicated that ptc-MIR171i-p3_1ss4TC, zma-MIR2275b-p3_2ss3CT17, vvi-miR403a_R + 1_1ss19TC and stu-miR397-5p_L + 2R-2_2 target *PLC4* and *BLH1*, *CBL10*, *AGO2* and *CRK2*, respectively. Among of these sweetpotato miRNAs, the level of ptc-MIR171i-p3_1ss4TC was significantly up-regulated in “Xushu 22” root compared “Xushu 32” roots under salt stress, while the levels of zma-MIR2275b-p3_2ss3CT17, vvi-miR403a_R + 1_1ss19TC and stu-miR397-5p_L + 2R-2_2 were significantly down-regulated in “Xushu 22” root compared “Xushu 32.” The previous study found that *blh1* mutants were less sensitive than the wild-type to salinity exposure during early seedling development. In contrast, *AtPLC4* and *BLH1* over-expressing lines were hypersensitive to salt stress (Kim et al., 2013; Xia et al., 2017). *AGO2*, *CRK2* and *CBL10* mediates salt tolerance in plants (Kim et al., 2007; Hunter et al., 2019; Wang et al., 2019; Yin et al., 2020). These results indicated that increasing the expression of their target genes *AGO2*, *CRK2* and *CBL10* by down-regulating zma-MIR2275b-p3_2ss3CT17, vvi-miR403a_R + 1_1ss19TC and stu-miR397-5p_L + 2R-2_2, and decreasing the expression of the target genes *PLC4* and *BLH1* by up-regulating ptc-MIR171i-p3_1ss4TC may be associated with better salt tolerance in “Xushu 22” than in “Xushu 32.”

In summary, our study generated comprehensive resources through a comprehensive analysis of transcriptome, sRNA and degradome sequencing data on sweetpotato, with the focus on the identification of key regulatory miRNAs in sweetpotato under salt stress. The results suggested that miRNA may play an important role in the salt stress response and adaptation of sweetpotato and thus may possibly result in the different salt sensitivity of different genotypes. The salt-responsive miRNAs and their target genes we identified can improve the understanding of the molecular mechanism of miRNA-mediated plant response to salt stress and can be utilized to optimize the salt stress resistance agronomic traits of sweetpotato in the future.

## Data Availability Statement

The datasets presented in this study can be found in online repositories. The names of the repository/repositories and accession number(s) can be found below: National Center for Biotechnology Information (NCBI) BioProject database under accession number: PRJNA600587.

## Author Contributions

TX and QC conceived and designed this manuscript. ZY, TD, XD, LZ, and MZ carried out the experiments and analyzed the data. ZY, TD, and TX wrote the manuscript. YW, YF, and GN helped to revise the manuscript. QC offered the plant material. All authors read and approved the manuscript.

## Conflict of Interest

The authors declare that the research was conducted in the absence of any commercial or financial relationships that could be construed as a potential conflict of interest.

## Publisher’s Note

All claims expressed in this article are solely those of the authors and do not necessarily represent those of their affiliated organizations, or those of the publisher, the editors and the reviewers. Any product that may be evaluated in this article, or claim that may be made by its manufacturer, is not guaranteed or endorsed by the publisher.

## Footnotes

1
http://unafold.rna.albany.edu/?Q=mfold

2
http://www.geneontology.org/
